# Principles of patient and public involvement in primary care research, applied to mental health research. A keynote paper from the EGPRN Autumn Conference 2017 in Dublin

**DOI:** 10.1080/13814788.2018.1470620

**Published:** 2018-05-31

**Authors:** Amanda Howe

**Affiliations:** Norwich Medical School, University of East Anglia, Norwich, Norfolk, UK

**Keywords:** Patient and public involvement, mental health research, change management

## Abstract

Clinical research relies on patients being willing to participate in research projects, and making this possible for patients with mental health problems can be a particular challenge. In the modern era, many countries have seen a movement to give a stronger voice to patients both in choices around their care and in how research is conducted. How to achieve effective patient and public involvement (PPI) and to make the patients real partners in this effort is itself a subject of research evaluation. This opinion piece—based on a keynote lecture given at the European General Practice Research Network 2017 autumn meeting in Dublin—describes both the reasons for expanding PPI, how it can usefully be achieved, and how this may relate to the particular context of mental health. There can be moral, methodological or policy reasons for PPI. The three commonest models of good practice in PPI are the ‘one off,’ the ‘fully embedded’ and the ‘outreach’ models. In research into common mental health problems in family practice, ‘outreach’ approaches that minimize commitment over time may work best. ‘Expert patients’ from mental health charities can sometimes play this role. PPI may be challenging and involve extra effort, but the gains for all may be considerable. Wonca Europe networks including EGPRN can extend this message and findings.

KEY MESSAGES

- Most funders of research in Europe recommend patient and public input (PPI) into research projects.
- PPI may be challenging and involve extra effort, but gains are considerable.
- Common models of good practice in PPI include the ‘one off,’ ‘fully embedded,’ and ‘outreach’ models—the latter particularly valuable for more vulnerable groups.

## Introduction

Most funders of research in Europe recommend patient and public input (PPI) into research projects, but there is little evidence on what this means in contemporary practice. Recent developments and evidence on PPI involves much more than the usual ‘consenting’ of adults for participation in research: it recommends a high level of involvement, where members of the public actively inform the research process from priority setting right through to methodological approaches and evaluation of the implications of findings. For example, the UK National Institute for Health Research describes PPI as being done ‘with’ or ‘by’ members of the public, rather than research being done ‘on’ or ‘to’ subjects—an active relationship, rather than a passive object [1].

Some studies have shown that researchers themselves are unaware of the potential of PPI to improve the quality and products of research, without using the full scope of possibilities that a partnership with the public may offer. Maximizing PPI may be even more challenging when the focus of the research is mental health (MH), as the population under study are more vulnerable and may have less capacity to engage.

This paper set out some context for the discussions that occurred during the EGPRN 2017 autumn meeting, looking at the definitions of PPI, its rationale, barriers and facilitators; and explores best practice in how to approach this as researchers, making some suggestions for getting PPI right in MH research.

## Reasons for patient and public input in research

Reasons for PPI in research can be grouped into:

- Moral reasons—it is the right thing to involve citizens in all activities that affect them, and to give them some choice and influence both over what research is funded and how it is done. The principle here is often expressed as ‘nothing about me without me’.
- Methodological reasons—health services research involves getting data from people in lived situations, so it is important to understand what may make them willing to consent to participate. PPI can help with the use of the right language, refining what is acceptable to patients in terms of the demands on their time or level of risk, and advice on how best to communicate and so recruit.
- Policy reasons—having effective PPI can influence the quality, outputs, and impacts of the work, as patients and organizations get more engaged, recruitment works, and groups feel more ownership for the results.

The author has experience of setting up an infrastructure for PPI in research, and has published some evaluations of this network, which trains volunteers to be prepared to advise research teams, and to join projects for longer-term inputs as needed [2–4]. She also drew on the findings of the UK national RAPPORT study (Research with patient and public involvement: a realist evaluation) [5,6], which examined multiple research projects to describe the workings of PPI.

## Models of good practice

Based on the evaluation literature, there appear to be three common models of PPI, (a) a ‘one off’ limited input, where a specific PPI input occurs with little preparation or involvement; (b) a ‘fully embedded’ model, where there is PPI with continuity of personnel throughout a project or programme; and (c) an ‘outreach’ model, where a particular community or understanding is needed—the last of these is also intermittent, but contact is ‘in depth’ and purposive.

Some principles of good practice in PPI found in the evaluation studies cited earlier include clear roles being agreed between researchers and the PPI leads: support for volunteers’ costs; time to build relationships of mutual respect; training for both groups to learn skills of enabling and supporting public involvement in research; communications in appropriate style and format; and full acknowledgement of the PPI in all reports. Having a named link for the PPI leads so they can access advice and clarify issues also seems to be an effective way of maximizing their impact and inputs (see Table 1 for some examples).

**Table 1.** Examples of three models for patient and public involvement (PPI), applied to mental health (MH) research.

PPI model	Example
(a) ‘One off’	A researcher, who is interested in how patients with psychosis access health services, arranges to attend a group therapy meeting at the local MH unit, and to talk with consented patients about these issues in a focus group following the therapy session. The researcher meets the five adults who agree only once, and sends them a summary of the findings when the project is written up.
(b) ‘Fully embedded’	A university recruits members of the public to advise on their health research. All volunteers receive training about research and how it is developed and conducted. They have a named member of staff who they can link up to for all activities, and each project they join also names a lead for their contact. Two of these PPI volunteers join the MH research team, and review all projects before they are funded, join an advisory group as projects go forward, and comment at all stages. One of them also trains to collect data by interviewing participants.
(c) ‘Outreach’	A research team wants to work with the mentally ill who have become homeless, but they know that this population will be challenging both to contact and to retain. They use local charities and community networks to find out who has regular contact with this population—then spending time and money to develop ways that these key groups (who are a trusted resource) can help to conduct the research in a way that is safe for the homeless and vulnerable, and that reflects their needs and barriers to support. In the process, the research design is radically altered by the views and inputs of the local community workers.

## Achieving change

There may be different drivers towards expanded PPI: if research funders, for example, expect to see evidence of PPI in new bids for grant funding, research institutions are likely to commit to such a change. But simply knowing that you ‘ought to’ or ‘have to’ do something does not always result in a good outcome. Any individual, research unit, or institution that aims to extend its capacity for effective PPI will need to go through a change process that shifts the whole team’s perspective and thinking. Findings from the RAPPORT study reflected the four stages of Carl May’s ‘Normalization Process Theory’ [7], which shows that people need to make sense of why a new approach may be needed, then develop some ownership of the approach; move into trying it out/piloting how best to make it work; and then adapting and reflecting on it until it becomes part of ‘normal’ life. Presenting evidence and reflecting on previous experiences or examples may help people to understand better what PPI can offer, followed by experimenting and evaluating until the change is accepted. A champion with previous experience in the field can be helpful, to show how they achieved such a change, and how it has added value; preferably offering objective critical evidence. Arguments for resourcing will also be needed, as there are expenses in terms of both people’s time and absolute costs of attendance, training; and sometimes salaries, if a PPI coordinator is appointed to assist this process on an ongoing basis within a research unit.

## Implications for MH research

In addition, MH research may raise particular challenges for PPI, so may benefit from different designs that allow patients and PPI leads to participate in ways that are safe and acceptable for them. Patients like ‘Billy’ with major MH diagnoses and adverse social environments or addiction problems are not likely to sit on research committees [8]. Health professionals may also be concerned about the additional burden of research, and so exclude patients from being approached for research studies. Even the common MH problems in family practice, such as psychological stress related to life events, may make active engagement in research and PPI activities seem like an added burden; and fluctuation in mental state can alter insight, or raise concerns about loss of confidentiality and stigma. The implications for PPI are that model (c) in Table 1—using outreach approaches that minimise commitment over time—may work better for this population. ‘Expert patients’ from MH charities can sometimes play this role, and the principles outlined earlier are still valid and important, but additional flexibilities and support are crucial in MH research.

## Sharing expertise for better MH research

In reflection on the author’s own ‘journey’ with PPI and research, I see strong parallels with the needs of the clinical consultation—a real commitment to the other person, who may be in a less powerful place and have different needs from your own; an attitude of flexibility and respect for diversity; a will to overcome bureaucratic and systemic barriers to achieve the best outcomes; a desire to use every situation to empower; and an awareness of interpersonal dynamics. There is also, of course, a need to retain a strong sense of the original research question and its scientific underpinnings—which PPI volunteers can both appreciate and help to develop.

In the context of MH research, some of the key areas include the long-term consequences of adverse life events, helping to prevent lifestyle risks such as addiction, and managing unexplained physical symptoms. These kinds of research need to happen in primary care settings where the patients are largely being cared for by family doctors. So it is for us and our practices to assist this involvement in MH research, and having patient advocates for such work may be very helpful.

Finally, it is worth noting the other networks that might be used to share and shape understanding of the issues under scrutiny. This article originated in discussion with the EGPRN, and also through the broader networks available through Wonca (World Organization of Family Doctors)—including academic members in university settings, and the Wonca Working Parties on Research, and on Mental Health. There are opportunities to add impact from our research findings by dissemination these through these networks, and also through the work of Wonca with the World Health Organization—where strong evidence can be built into global strategy. The principles of good practice can also inform PPI in local situations, such as research networks, or even in clinical practice, as many of the key findings, which make a productive and equal partnership, may not be specific to the research context.

## Conclusion

The trend towards a stronger voice for patients in their own care and experiences is mirrored in research where giving the public more opportunity to shape what research is done and how it is conducted can enhance the quality of evidence available to us. While this may be challenging and involve extra effort the gains for all may be considerable. Family doctors and their academic leads are well placed to help patients, including those more vulnerable such as MH sufferers, to engage with research: and our clinical method of person-centred care with empowerment of the individual lends itself to a more empowered relationship with lay volunteers in research. Wonca networks including EGPRN can extend this message and findings.
